# Severe acquired cytomegalovirus infection in a full-term, formula-fed infant: Case Report

**DOI:** 10.1186/1471-2431-11-52

**Published:** 2011-06-06

**Authors:** Alberto Berardi, Cecilia Rossi, Valentina Fiorini, Cristina Rivi, Federica Vagnarelli, Nicola Guaraldi, Paola Pietrosemoli, Tiziana Lazzarotto, Fabrizio Ferrari

**Affiliations:** 1 Unità Operativa di Terapia Intensiva Neonatale, Azienda Ospedaliero-Universitaria Policlinico di Modena, Italy; 2 Unità Operativa di Pediatria, Ospedale Ramazzini di Carpi, Italy; 3 Unità Operativa di Terapia Intensiva Neonatale, Arcispedale Santa Maria Nuova, Reggio Emilia, Italy; 4 Unità operativa di Pediatria, Azienda Ospedaliero-Universitaria Policlinico di Modena, Italy; 5 Struttura Complessa di Microbiologia e Virologia, Azienda Ospedaliero-Universitaria Policlinico di Modena, Italy; 6 Unità Operativa di Microbiologia, Azienda Ospedaliero-Universitaria Policlinico S. Orsola Malpighi, Università di Bologna, Italy

## Abstract

**Background:**

Cases of cytomegalovirus colitis are exceptionally reported in immuno-competent infant. The pathogenesis is uncertain but breast-feeding is considered as a main source of postnatal infection.

**Case Presentation:**

Here we report a full-term, formula-fed infant who developed a severe cytomegalovirus anaemia and colitis when aged 2 months.

**Conclusion:**

Even if the molecular identity between the cytomegalovirus-isolate of the infant and the maternal virus could not be demonstrated, we confirmed through laboratory investigation that cytomegalovirus infection was acquired postnatally. However, the source of cytomegalovirus infection remained unclear. Alternative modes of cytomegalovirus transmission are discussed.

## Background

Cytomegalovirus (CMV) is a ubiquitous agent that determines a wide spectrum of diseases in children and adulthood. Severe cytomegalovirus infection is often reported when disease is congenital [1], acquired in premature infants [2] or in immuno-compromised patients.

Cases of CMV gastrointestinal disease have been exceptionally observed in immuno-competent infants. Congenital origin could not be ruled out in all reported cases. Infants were breast fed and most of them presented CMV-disease between 5 to 8 weeks after birth, with severe diarrhoea and fever. Possibly, the CMV transmission occurred at birth or soon after, as the incubation period of CMV infection generally ranges between 4 and 12 weeks.

Risk factors and pathogenesis of CMV gastrointestinal disease which affects term infants during the first months of life are poorly defined.

## Case Presentation

A Chinese male infant was delivered at term by emergency caesarean section because of foetal distress. The mother was at first pregnancy and presented with membrane rupture 24 hours before delivery. Maternal CMV primary infection was diagnosed at the 27^th ^week of gestation (negative serum specific CMV IgG in early pregnancy. Positive serum specific CMV IgM at 27^th ^weeks of gestation. Positive CMV IgG antibodies 2 weeks later).

Family pedigree was negative for atopy, immuno-deficiencies and gastrointestinal diseases. Birth weight was 3255 g, cranial circumference 34 cm and length 51 cm; APGAR score was 9 and 10 at the 1^st ^and 5^th ^minute respectively. An urine polymerase chain reaction (P.C.R.) was CMV negative at birth.

Fourteen hours after birth the newborn presented with a severe abdominal distension and breast feeding was soon and permanently discontinued. When 2 days old the newborn was referred to our neonatal intensive care unit because of acute intestinal occlusion and ileum-cecal valve atresia. Two consecutive urine shell vial assays, obtained after admission, were CMV early antigen negative as like as specific serum CMV-IgM antibodies. In the 4^th ^day of life the baby underwent intestinal resection. No blood products were administered and no inclusion bodies were found in the intestinal biopsy. Three weeks later the baby was discharged home; he was formula-fed and healthy appearing.

When aged 10 weeks the infant showed fever, irritability and decreased milk intake. Physical examination revealed a lethargic infant, pale-greyish skin, marbled limbs, severe hepato-splenomegaly and tachycardia. Blood analysis showed: white blood cell count 7.63 per 10^9^/L (31% neutrophils; 61% lymphocytes; 7% monocytes), haemoglobin 6.8 g/dl, hematocrit 21%, platelet count 181 per 10^9^/L, aspartate-aminotransferase 62 U/L, alanine-aminotransferase 39 U/L, albumin 1.8 g/dl, with normal clotting time test. Serum specific CMV immunoglobulin levels (IgG and IgM) were consistent with a primary infection.

The infant showed a clinical improvement by therapy (intravenous fluids, broad spectrum antibiotics, albumin and packed erythrocytes), but 6 days after admission he presented a severe haemorrhagic diarrhoea. No parasites were found by stool examination and *Rotavirus/Adenovirus *chromatographic immunoassay was negative. Stool cultures for *Salmonella, Shigella, Campylobacter *and *Yersinia *species were also negative.

A large intestine biopsy showed a chronic inflammation with lymphatic hyperplasia and colic crypt lesions.

Urine shell vial assay was CMV early antigen positive. P.C.R. was positive in the biopsy (quantitative CMV-DNA 2620 copies/100.00cells), in bone marrow (quantitative CMV-DNA 2275 copies/ml) and in peripheral blood (determined 7 days after start of antiviral therapy: quantitative CMV-DNA 240 copies/ml).

Brain ultrasound scans, cranial computed tomography, ophthalmologic examination and auditory brainstem responses were normal.

Intravenous ganciclovir (GCV) (10 mg/kg/day) was administered for 3 weeks. The diarrhoea improved after 3 days of GCV. Formula-feeding was rapidly increased and the baby was discharged 37 days after admission.

Detailed investigation did not reveal any underlying primary immunodeficiencies (normal lymphocyte subsets studies, serum immunoglobulin and IgG sub-class levels). Urinary shedding of CMV ceased during the second year of life and neuro-developmental outcome at 2 years of age is normal.

## Conclusions

In immuno-competent host, acquired CMV infection is usually asymptomatic or may determine a mild disease. Severe gastrointestinal disease can be observed in congenital infections [1], in children with AIDS, or in children receiving immunosuppressive therapy post-transplantation.

CMV may also cause relapses of intestinal disease in infants affected by inflammatory bowel diseases [3]. Additional sources are blood transfusions or contact with genital tract secretions at delivery, whereas viral shedding from maternal pharynx or urinary tract, during the first months after birth, is not associated with perinatal transmission [4].

Finally, breast feeding is a recognized source of CMV in the perinatal period and an important cause of "sepsis-like" infection in very preterm infants [2]. Cheong and co-workers [5] reported 11 cases of postnatal CMV infection with gastrointestinal manifestations. All infants were breast fed, 10 were delivered preterm (range 24 to 32 wks of gestation) and one, low birth weight newborn, was affected by Rothmund-Thomson syndrome. Premature newborn is an immunocompromised host and CMV gastrointestinal disease may result from enteral acquisition after infected fresh milk feeding.

In contrast, severe CMV gastrointestinal disease have been exceptionally reported in immunocompetent infants [6-10]. Congenital origin could not be ruled out, therefore the pathogenesis is still unclear. Common findings include breast feeding, the need of antiviral therapy (4 of 5 cases) and the onset of symptoms between 5 to 8 weeks after birth (Table 1). The incubation period of CMV infections generally ranges between 4 and 12 weeks; it is therefore likely that most of these infants acquired infection at or soon after birth.

**Table 1:** Severe gastrointestinal cytomegalovirus infection in immuno-competent infants born at term

Reference	Age at onset	Breast feeding	Clinical findings	Diagnosis	Mode of transmission	Antiviral therapy
**Jonkhoff-Slok (8)**	5 weeks	Yes°	Bloody diarrhoea Rectal bleeding	Inclusion bodies at intestinal biopsy	Perinatal ? Cow's milk allergy ?	No

**Fox (7)**	5 weeks	Yes	Fever, Lymphoadenopathy Intractable diarrhoea Occult blood in stools	Inclusion bodies at duodenum biopsy	Congenital ? Perinatal ?	i.v. GCV for 5 weeks

**Quiros-Tejeira (9)**	2 months	Yes	Fever, watery diarrhoea	Severe colonic inflammatory changes, inclusion bodies	Perinatal ?	i.v. GCV for 2 weeks

**Rongkavilit (10)**	8 weeks	Yes	Profuse watery diarrhoea	Inclusion bodies in duodenum and sigmoid colon	Perinatal ? Postnatal (breast-feeding ?)	i.v. GCV for 4 weeks

**Buonuomo (1)**	8 weeks	Yes	Fever Anaemia Melena §	Positive PCR at duodenum biopsy	Perinatal ?	Oral VGCV for 5 weeks

GCV = Ganciclovir; VGCV = Valganciclovir; PCR = polymerase chain reaction; i.v. = intravenous

° The infant was breast fed for 4 weeks, then he was formula fed

§ Melena occurred at the age of 7 months

The infant here described had no clinical signs of the disease at birth and laboratory testing for CMV (at birth) were negative, therefore we could firmly exclude congenital infection. No recognizable underlying illness or risk factor for CMV were found. Unlike previous reports, the infant was breast-fed for a few hours at birth, and then formula-fed.

Horizontal CMV transmission from another child in the household was unlikely, as the infant was an only child.

The mother, who suffered from primary infection in late pregnancy, was the likely source of CMV (perhaps at birth, as a consequence of prolonged membrane rupture). Perhaps intestinal surgery during the first week of life increased disease severity. We speculate that mucosal injury possibly led to increased permeability with a massive intestinal and blood CMV invasion. The hypothesis of mucosal injury is supported by the observation of CMV colitis in immunocompetent children with inflammatory bowel disease [3] or cow's milk allergy [8]. Further, the disease could have been worsened by the lack of breast milk and maternal protective factors (namely IgA antibodies).

Biopsies and P.C.R. assays confirmed severe bone marrow and intestinal CMV involvement, which promptly recovered after antiviral therapy.

This study has some limitations. Indeed, we could not demonstrate a molecular identity between the CMV-isolate of the infant and the maternal virus. Therefore the source of CMV remains speculative, though a transmission from the maternal source it is likely.

Further, no amniocentesis was performed and some information on maternal infection (DNAemia, viral shedding in urine or saliva) was lacking. The characterization of maternal primary infection during pregnancy was therefore incomplete.

Nevertheless, this case report contribute to understand CMV end-organ-disease and related symptomatic infection of term infants during the first months of life. It shows that severe disease may be acquired after birth, even in immunocompetent, formula-fed, full-term infant. Intractable diarrhoea may need ganciclovir therapy.

## Abbreviations

CMV: cytomegalovirus; P.C.R.: polymerase chain reaction; GCV: ganciclovir; VGCV: Valganciclovir.

## Competing interests

The authors declare that they have no competing interests.

## Authors' contributions

All authors contributed to conception and design, acquisition of data, analysis or interpretation of data and gave final approval of the version to be published. In detail:AB and FV drafted the manuscript; CR, VF, CR and NG conceived of the study, participated in its design and coordination and helped to draft the manuscript; PP and TL carried out the immunoassays and molecular studies; FF have been involved in revising the manuscript critically for important intellectual content.

## Pre-publication history

The pre-publication history for this paper can be accessed here:

http://www.biomedcentral.com/1471-2431/11/52/prepub
